# A Red-Light-Responsive DASA–Polymer with High Water Stability for Controlled Release

**DOI:** 10.3390/polym15112489

**Published:** 2023-05-28

**Authors:** Hao Ma, Wan Li, Haojun Fan, Jun Xiang

**Affiliations:** College of Biomass Science and Engineering, Sichuan University, Chengdu 610065, China

**Keywords:** red light, photoresponsive polymers, donor-acceptor Stenhouse adducts, nanovectors, drug release

## Abstract

Photoresponsive polymers hold vast potential in the realm of drug delivery. Currently, most photoresponsive polymers use ultraviolet (UV) light as the excitation source. However, the limited penetration ability of UV light within biological tissues serves as a significant hindrance to their practical applications. Given the strong penetration ability of red light in biological tissues, the design and preparation of a novel red-light-responsive polymer with high water stability, incorporating the reversible photoswitching compound and donor-acceptor Stenhouse adducts (DASA) for controlled drug release is demonstrated. In aqueous solutions, this polymer exhibits self-assembly into micellar nanovectors (~33 nm hydrodynamic diameter), facilitating the encapsulation of the hydrophobic model drug Nile red (NR) within the micellar core. Upon irradiation by a 660 nm LED light source, photons are absorbed by DASA, leading to the disruption of the hydrophilic–hydrophobic balance of the nanovector and thereby resulting in the release of NR. This newly designed nanovector incorporates red light as a responsive switch, successfully avoiding the problems of photodamage and limited penetration of UV light within biological tissues, thereby further promoting the practical applications of photoresponsive polymer nanomedicines.

## 1. Introduction

In recent years, stimulus-responsive nanomedicines have become an important direction for the development of advanced nanomedicines, owing to their capacity to achieve enhanced drug accumulation and on-demand drug release at the targeted cancer tissues, thereby considerably decreasing drug dosage and toxicity [1]. So far, pH-[2], redox-[3], photo-[4,5], and temperature-responsive [6] nanomedicines have been developed. Of these, nanomedicines constructed from photoresponsive polymers have garnered significant attention from researchers owing to their distinctive advantages. On the one hand, remote and precise control of drug release can be achieved by controlling the opening or closing of the light source, providing immense convenience for regulating the drug concentration within the therapeutic window [7]. On the other hand, their drug release is independent of the cancer tissues’ microenvironment, enabling the use of the same nanomedicine to treat cancer in various microenvironments, thus rendering them highly versatile [8,9,10].

To construct photoresponsive polymer nanomedicines, researchers often incorporate light-responsive compounds, such as azobenzene [11], *o*-nitrobenzyl ester [11,12,13], coumarin [14,15], and spiroxamine derivatives [16], into the molecular structure of polymer chains. However, their responsive wavelengths are generally in the ultraviolet (UV) region, necessitating the use of UV light sources for drug release of the corresponding nanomedicines. It is well-known that UV photons have high energy and can cause phototoxicity to cells and biological tissues, which greatly limits the practical application of photoresponsive polymer nanomedicines [17]. Given the strong penetration depth and low toxicity of visible light in biological tissues, the design and preparation of photoresponsive polymer nanomedicines integrated with visible light-responsive compounds have become a research hotspot in this field [17,18,19].

Donor-acceptor Stenhouse adducts (DASAs) were initially discovered as visible light-responsive compounds in 2014 [20]. They offer several advantages, such as strong molecular designability [21,22,23], tunable responsive wavelengths [24,25], and low toxicity [26], and have demonstrated broad application prospects in polymer actuators [24,27], drug release [4,22,28], and food safety warning [29]. Over the past few years, the exploration of DASA-based photoresponsive polymers has extended to their potential use as nanovectors [4,28,30,31]. Usually, when exposed to visible light, the embedded DASA units within them undergo an isomerization process, transitioning from the hydrophobic triene form to the hydrophilic cyclopentenone form. This transformation disrupts the hydrophilic-to-hydrophobic equilibrium, subsequently initiating the release of the encapsulated drug.

**Scheme 1:**
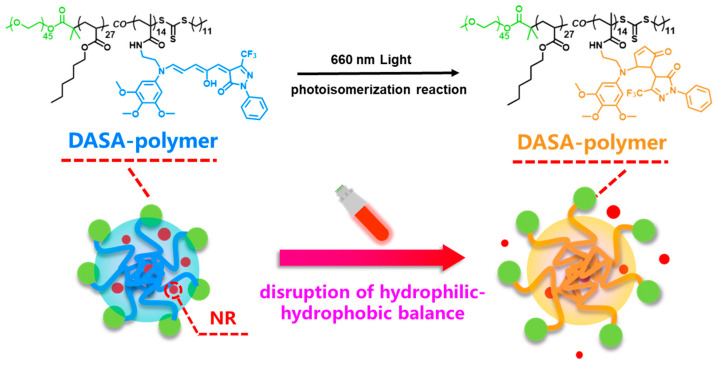
Schematic illustration of the red light-induced release of a model drug, Nile red (NR), from the DASA–polymer nanovector resulting from the isomerization reaction of grafted DASA units.

It is important to note that in addition to light, the presence of water molecules in biological tissues can significantly influence the isomerization of DASA units, resulting in the undesired leakage of drug molecules [4,31,32]. This highlights the need for the consideration of water stability when developing DASA-based photoresponsive polymers for efficient and controlled drug release. Despite much progress, the number of suitable DASA-based polymers remains limited. Therefore, this work presents a novel design and synthesis of DASA-based polymers with high water stability, utilizing the strong penetrating ability of red light [18,33,34] to achieve photocontrolled drug release (Scheme 1).

In the subsequent sections, we first demonstrate the successful synthesis of the designed compounds and the red-light-responsive DASA–polymer. Subsequently, we investigate its assembly morphology in water and examine the influence of water on the isomerization of DASA within the DASA–polymer assemblies before and after irradiation. Finally, using the model drug Nile red (NR), known for its extensive use in drug release studies [35,36,37,38], we aim to illustrate the potential of our designed DASA–polymer as a platform for photocontrollable drug release. As shown below, the combination of the strong penetration capability of red light and the good water stability of DASA-based photoresponsive polymer nanovector not only advances the field of DASA-based nanovectors but also promotes the practical application of photoresponsive polymer nanomedicines.

## 2. Materials and Methods

### 2.1. Materials

*2*-furaldehyde (99%), acetic acid (99%), ethanol (99%), NaOH (98%), potassium carbonate (≥99.5%), anhydrous sodium sulfate (Na_2_SO_4_, ≥99%), methanol (99.9%), pentafluorophenol (99%), methacryloyl chloride (95%), *2*,*6*-dimethylpyridine (99%), *2*,*2*-azobis(2-methylpropionitrile) (AIBN, ≥98%), *n*-hexyl acrylate (HA, ≥98%), dichloromethane (DCM, ≥98%), sodium bicarbonate (NaHCO_3_, ≥99.5%), sodium chloride (NaCl, ≥99.5%), anhydrous magnesium sulfate (MgSO_4_, ≥98.0%), *1*,*4*-dioxane (99.7%), methanol (99.9%), ethanol (≥99.7%), tetrahydrofuran (THF, 99%), *N*, *N*-dimethylformamide (DMF, 99%), triethylamine (TEA, 99%) were supplied by Kelong Chemical Engineering Co. Ltd. (Liaoning, China). Phenylhydrazine (99%), ethyl *4*,*4*,*4*-trifluoroacetoacetate (98%), (*L*)-proline (99%), *3*,*4*,*5*-trimethoxyaniline (≥98%), *N*-(*2*-bromoethyl)phthalimide (≥98%), concentrated hydrochloric acid (37%), ethyl ether (≥99.7%), hydrazine hydrate (50–60%) were supplied by Adamas-beta Reagent Co., Ltd. (Shanghai, China) Nile red (NR, ≥95.0%) was obtained from Macklin. Pluronic F-127, poly(ethylene glycol) methyl ether (PEO_45_-OH, *M*n = 2 kg mol^−1^), and other reagents were purchased from Sigma-Aldrich Co. AIBN was purified by recrystallization before use. The RAFT agent *S*-dodecyl-*S′*-(α,α′-dimethyl-α″-acetic acid)trithiocarbonate (DDMAT) and macromolecular PEO_45_-DDMAT were synthesized as previously reported [39,40]. Ultrapure water was obtained from a Millipore Simplicity 185 system at 18.2 MΩ·cm.

### 2.2. Instruments and Measurements

Recordings of ^1^H, ^19^F, and ^13^C NMR spectra were conducted using a 400 MHz Bruker AV III HD spectrometer at 25 °C with tetramethylsilane as the internal standard. The mass spectra were obtained using a Q-Exactive plus mass spectrometer (Thermo Fisher Scientific, Waltham, MA, USA). Gel permeation chromatography (GPC) measurements were taken at 20 °C with a Waters 1515 separation module equipped with a Waters 2707 automatic sampler and a Waters 2414 refractive index detector. The eluent used was THF at a flow rate of 1 mL min^−1^. The molecular weight of polymers was calculated according to the polystyrene standard using Waters Millennium module software. UV–Vis measurements were performed using a 3900H Hitachi UV-visible spectrophotometer. Fluorescence spectra were recorded with a Fluorescence Spectrophotometer F-7100 at 25 °C. To obtain transmission electron microscopy (TEM) images, samples were prepared by dropping the sample solution (~15 μL) on the surface of carbon-coated copper grids (Beijing XXBR Technology Co., Ltd., Beijing, China, T11023). After solvent evaporation, TEM measurements were conducted using an HT7700 Hitachi microscopy at an acceleration voltage of 80 kV. Dynamic light scattering (DLS) experiments were performed with a Malvern Zetasizer Nano ZS ZEN3600 system equipped with a helium-neon laser (λ = 633 nm) at a scattering angle of 173°. To prepare the samples for this test, 1.25 mg of DASA–polymer was dissolved in 2.5 mL of tetrahydrofuran (THF). While vigorously stirring the THF solution, 10 mL of ultrapure water was slowly added at a rate of 1 mL min^−1^. The stirring process was conducted in a dark environment for 2 h. The THF was subsequently removed using rotary evaporation, resulting in the formation of a red-light-responsive micellar aqueous solution. Next, 0.5 mL of the prepared solution was diluted five fold with ultrapure water, resulting in a concentration of 0.025 mg mL^−1^. The diluted solution was then sonicated for 10 seconds. The resulting diluted samples were subjected to DLS measurements before and after illumination. It is important to note that no additional salts or substances were introduced during the preparation process, and the pH was maintained at 6.5. The LED light source (λ = 660 nm) used in the experiment was purchased from Guangzhou Hangxin Photoelectric Technology Co., Ltd. (Guangzhou, China), and the power intensity of this LED light was determined by a CEL-NP2000 full-spectrum high-light optical power meter (Beijing Education Au-light Co., Ltd., Beijing, China).

### 2.3. Synthesis Procedures

**Scheme 2:**
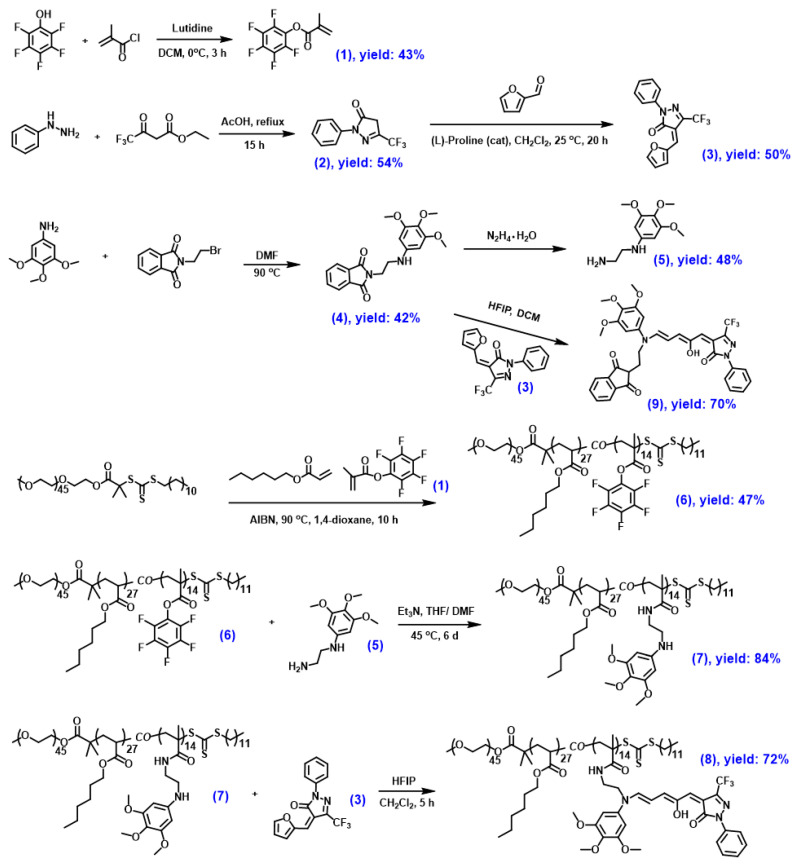
The synthetic route for DASA–polymer.

The synthetic route of the DASA–polymer is shown in Scheme 2. Details are described as follows.

#### 2.3.1. Synthesis of Compound (**1**)

Compound (**1**) was synthesized according to our previously published work [4]. ^1^H, ^13^C, and ^19^F NMR spectra of compound (**1**) are presented in Figure S1.

#### 2.3.2. Synthesis of Compounds (**2**) and (**3**)

Compounds (**2**) and (**3**) were synthesized according to a previously published paper [41]. ^1^H NMR spectra of compounds (**2**) and (**3**) are presented in Figure S2.

#### 2.3.3. Synthesis of Compound (**4**)

A round-bottom flask was charged with 8.8 g *3*,*4*,*5*-trimethoxyaniline, 12.2 g *1*-bromo-*2*-phthalimidoethane, 6.6 g K_2_CO_3_, and 12 mL DMF. After stirring at 90 °C for 20 h, the resulting solution was poured into 900 mL cold H_2_O and kept in a fridge for 2 h. After obtaining the brownish-yellow precipitated solid by filtration, this solid was further washed with cold H_2_O three times and dried at 40 °C in a vacuum oven overnight. Finally, the product was purified by recrystallization in ethanol to obtain a yellow compound (**4**) (yield 8.9 g, 42%). The ^1^H NMR and mass spectra of compound (**4**) are presented in Figure S3.

#### 2.3.4. Synthesis of Compound (**5**)

First, compound (**4**) (2.0 g) was dissolved in 200 mL boiling ethanol. Second, 0.25 mL hydrazine hydrate was dropwise added to it and the whole solution was heated to reflux for 4 h, during which a white precipitate was produced. Third, the resulting solution was cooled to room temperature and its pH was adjusted to 3 by adding concentrated HCl. After 1 h, the precipitate was filtered off and the filtrate was concentrated to half its original volume using a rotary evaporator. The pH was adjusted to 10 by the addition of NaOH and extracted five times with ether. The organic phase was combined and dried over Na_2_SO_4_, filtered, and concentrated using a rotary evaporator. Finally, the product was dried at 40 °C in a vacuum oven overnight to offer compound (**5**) (*N*^1^-(*3*,*4*,*5*-trimethoxyphenyl)ethane-*1*,*2*-diamine, TMPEDA) and it was used in the following experiment without further purification. The ^1^H NMR and mass spectra of compound (**5**) (yield 0.61 g, 48%) are presented in Figure S4.

#### 2.3.5. Synthesis of Polymer (**6**)

**Figure 1:**
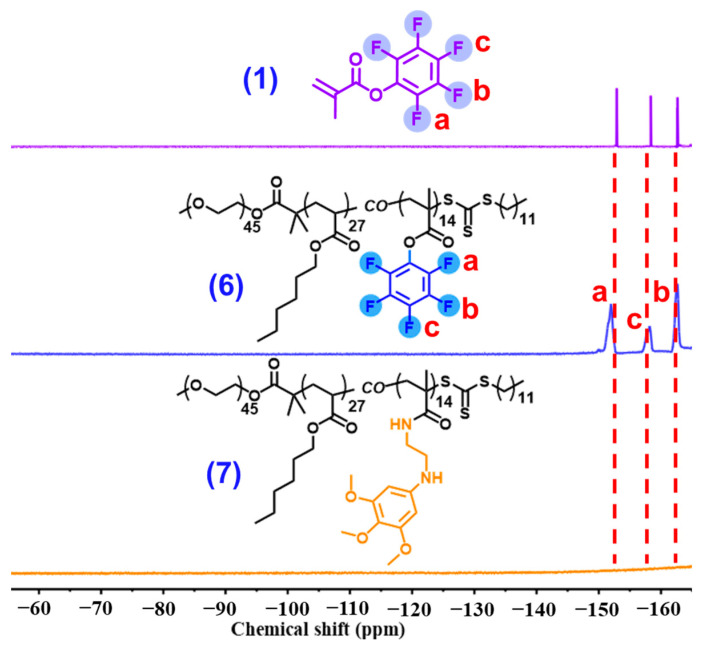
^19^F NMR (25 °C, 400 MHz) spectra of compound (**1)** (**top**), polymer (**6**) (**middle**), and polymer (**7**) (**bottom**) in CDCl_3_.

Polymer (**6**) (PEO_45_-*b*-P(PFPMA_14_-*co*-HA_27_)) was synthesized according to our previously published work [4]. Briefly, 500 mg PEO_45_-DDMAT macroinitiators, 4.096 mg AIBN, 1.649 g compound (**1**), and 1.028 g HA were dissolved in 1.15 mL *1*,*4*-dioxane. The solution was degassed by bubbling N_2_ gas. Subsequently, the sealed mixture was heated at 90 °C for 10 h. Finally, the mixture was precipitated in 60% methanol/water to obtain polymer (**6**) (yield 1.51 g, 47%). ^1^H and ^19^F NMR spectra of (**6**) are presented in Figure S5 and Figure 1 (middle), respectively.

#### 2.3.6. Synthesis of Polymer (**7**)

**Figure 2:**
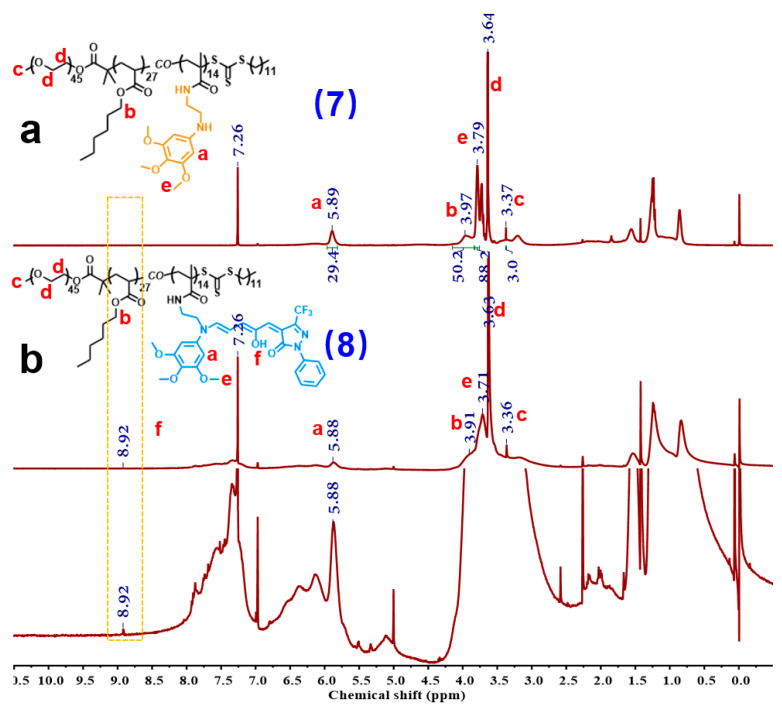
^1^H NMR (25 °C, 400 MHz) spectra of (**a**) polymer (**7**) and (**b**) DASA–polymer (polymer (**8**)).

First, 0.414 g polymer (**6**) was dissolved in a solution of 9 mL THF and 2 mL DMF. Then, the compound (**5**) and 191 μL TEA were added to this solution. This mixture was stirred at 45 °C for 7 days. After removing the precipitated solid by centrifugation, the whole solution was dialyzed against THF for 6 days (MWCO = 3500 Da) to remove small-molecule species and then recovered with a rotary evaporator. Finally, a brown solid (polymer (**7**), (PEO_45_-*b*-P(TMPEDA_14_-*co*-HA_27_))) was obtained. ^19^F NMR and ^1^H spectra of polymer (**7**) are presented in Figure 1 (bottom) and Figure 2, respectively.

#### 2.3.7. Synthesis of DASA–Polymer (Polymer (**8**))

**Figure 3:**
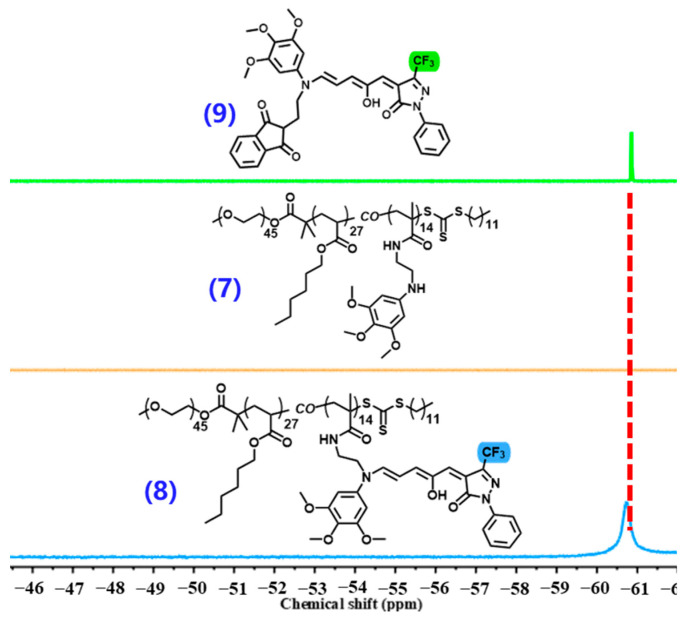
^19^F NMR (25 °C, 400 MHz) spectra of compound (**9**, DASA), polymer (**7**), and DASA–polymer (polymer (**8**)).

First, 250 mg polymer (**7**) was dissolved in 1.5 mL THF. Then, 300 mg compound (**3**) and 0.38 mL *1*,*1*,*1*,*3*,*3*,*3*-Hexafluoroisopropanol (HFIP) were added to this solution, and the reaction was performed at room temperature for 5.5 h under magnetic stirring. To remove excess compound (**3**), this mixture was dialyzed against THF for 7 d and then recovered by a rotary evaporator. Finally, a blue solid was obtained. ^1^H and ^19^F NMR spectra of polymer (**8**) (PEO_45_-*b*-P(DASA_14_-*co*-HA_27_)), namely, DASA–polymer, are presented in Figure 2 and Figure 3, respectively.

#### 2.3.8. Synthesis of Compound (**9**)

Next, 98 mg compound (**3**), 114 mg compound (**4**), and 0.2 mL HFIP were dissolved in 0.8 mL CH_2_Cl_2_. After reaction at 25 °C for 1 h, CH_2_Cl_2_ and HFIP were removed under reduced pressure. The remaining solid was triturated in diethyl ether. After filtration, compound (**9**) was isolated as a royal purple solid (0.145 g, yield 70%). ^1^H, mass, and ^19^F NMR spectra of compound (**9**) are presented in Figure S6 and Figure 3, respectively.

### 2.4. Preparation of DASA–Polymer Micelles

To prepare DASA–polymer micelles, we added 1.25 mg DASA–polymer into 10 mL THF. Under intense stirring, this solution was added dropwise into 10 mL of ultrapure H_2_O. The solution was then stirred in the dark for a period of 2 h. Upon removal of THF through rotary evaporation, the solution was stored in the dark at room temperature until it was ready to be utilized.

### 2.5. Preparation of NR-Loaded DASA–Polymer Nanovectors

To prepare the NR-loaded nanovector, we added 0.125 mg NR into a 2.5 mL THF solution containing 1.25 mg DASA–polymer. Under intense stirring, this solution was added dropwise into 10 mL of ultrapure H_2_O. Subsequently, the solution was stirred in the absence of light for a duration of 2 h. Upon removal of THF through rotary evaporation, the resulting solution underwent centrifugation at 4000 RPM for a duration of 15 min. Lastly, the supernatant was subjected to filtration via a 220 nm hydrophilic PTFE filter to acquire the intended nanovector. This sample was stored at room temperature under dark conditions prior to use.

## 3. Results and Discussion

Before synthesizing polymers, we first determine whether the compounds (**1**)–(**5**) are successfully prepared using NMR and mass spectroscopy. Their ^1^H, ^13^C, ^19^F NMR, and mass spectra are shown in Figures S1–S4. Our results indicate that they have been successfully synthesized.

To finally obtain DASA–polymer, we need to introduce secondary amine groups into the amphiphilic block polymer, so that it can further undergo a ring-opening reaction with compound (**3**) to gain DASA–polymer (polymer (**8**) in Scheme 2). First, according to our previous work [4], the amphiphilic block polymer of polymer (**6**) containing pentafluorophenyl ester was synthesized by RAFT polymerization. Its ^1^H NMR spectrum is shown in Figure S5. It can be seen that the hydrogen protons peaks of CH_3_-, -CH_2_- in CH_3_-O-CH_2_-CH_2_- appeared at 3.37 and 3.64 ppm, respectively. These data indicate that *n*-hexyl acrylate (HA) has been successfully incorporated into polymer chains. By calculation, the number of repeating HA units in the polymer (**6**) is 27 based on the data shown in Figure S5.

Next, we demonstrated the successful introduction of pentafluorophenyl methacrylate (PFPMA, compound (**1**) in Scheme 2) into the polymer (**6**) by ^19^F NMR spectroscopy. As shown in Figure 1, the peaks appeared at −152.89, −158.34, and −162.63 ppm directly prove that fluorine-contained moieties, namely, pentafluorophenyl ester, have been successfully inserted into the polymer backbone.

**Figure 4:**
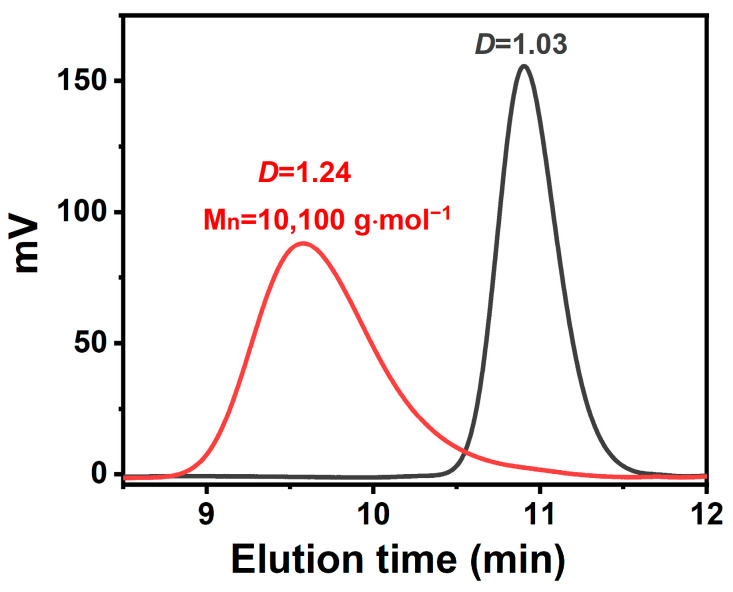
GPC traces of polymer (**6**) (red) and PEO_45_-DDMAT (black).

To analyze the number of repeating PFPMA units, we carried out GPC tests on the polymer (**6**) and PEO_45_-DDMAT (Table S1). As shown in Figure 4, the number average molecular weight (*M*n) of polymer (**6**) is 1.01 × 10^4^ g mol^−1^ and PDI is 1.24. Combined with the GPC results, the number of repeating HA units, and the *M*w of PEO_45_-DDMAT (2346 g mol^−1^), the number of repeating PFPMA units in the polymer (**6**) is 14.

Next, the amphiphilic block polymer having secondary amines was prepared using compound (**5**) to react with polymer (**6**). The ^19^F NMR spectrum of the product, polymer (**7**), is given in Figure 3. As shown, three peaks (a, b, and c) in the ^19^F NMR spectrum of polymer (**6**) disappear, indicating the degree of substitution reaches 100%.

Similarly, the compositional analysis of polymer (**7**) was performed using ^1^H NMR spectroscopy. As shown in Figure 2a, the typical peaks appear at 3.79 and 5.89 ppm indicating that moieties have been successfully introduced. Furthermore, based on the integrated area of the peak observed at 3.79, it can be deduced that the number of repeating secondary amine units in the polymer (**7**) is 14.7, which also indicates the degree of substitution reaches 100%. All above data verify polymer (**7**) has been successfully prepared.

Finally, DASA–polymer was prepared by reacting polymer (**7**) with compound (**3**), and the result of ^1^H NMR spectroscopy is shown in Figure 2b. Although the peaks are complex, we still found a characteristic peak attributable to the proton in the hydroxyl group of the linear DASA units (8.92 ppm; the magnified ^1^H NMR spectrum of polymer (**8**), located at the bottom of Figure 2b, provides greater clarity of this peak), which is almost located at the same position that of the model DASA small molecule we designed and synthesized (8.91 ppm, see Figure S6), indicating that the DASA units have been successfully introduced into the amphiphilic block copolymer.

To further confirm the successful incorporation of DASA, we noticed the presence of fluorine atoms in the DASA unit and therefore performed ^19^F NMR spectroscopy. As shown in Figure 3, both DASA–polymer and compound (**9**) exhibited a peak at around -61 ppm, while the polymer (**7**) did not show any peaks at the same position. The results also indicated that DASA units have been successfully incorporated into the amphiphilic block copolymer.

**Figure 5:**
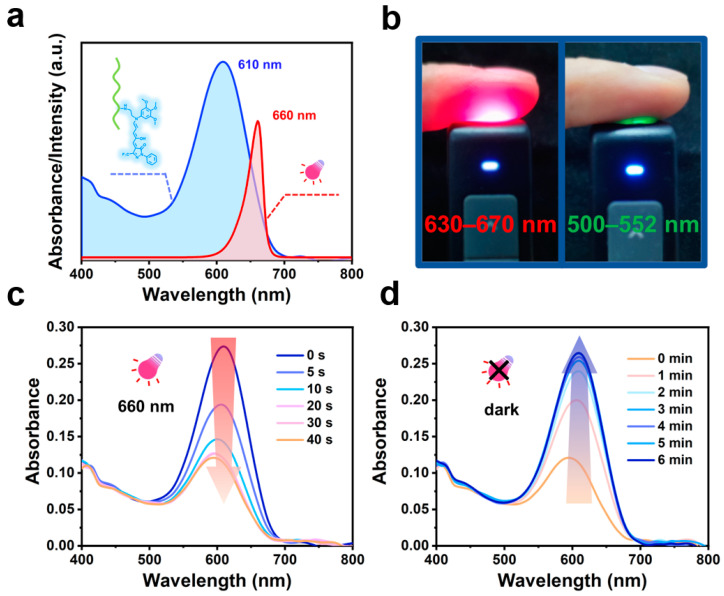
(**a**) UV–Vis spectra of DASA–polymer (solid blue line) in THF and emission spectrum of the red light LED. (**b**) In contrast to green light, the image demonstrates the potent penetration capacity of red light on the human finger. Time-dependent absorption spectral changes of DASA–polymer in THF under 660 nm irradiation (150 mW cm^−1^, (**c**)) and its subsequent thermal recovery in the dark (**d**).

Next, we investigated the optical properties of DASA–polymer in THF. As shown in Figure 5a, DASA–polymer displays a broad absorption peak in the 500–700 nm region with a maximum absorption peak at 610 nm. As this absorption peak partially overlaps with the emission band of a 660 nm LED, we hypothesized that irradiation with this LED light should initiate the photoisomerization reaction. It is worth noting that we chose the 660 nm LED light source because it has better penetration capability than green light in biological tissue (Figure 5b).

Figure 5c shows the changes in the UV–Vis absorption spectrum of DASA–polymer after exposure to 660 nm. It is evident that the absorbance of the maximum absorption peak gradually decreases with increasing irradiation time. The initial decrease was large within the first 20 s, but the rate of decrease gradually slowed thereafter. As the photochemical reaction of DASA is reversible, the colorless DASA chromophore should revert to its original-colored form upon cessation of light irradiation under dark conditions. As expected, when the irradiated DASA–polymer solution was placed in the dark, we observed a gradual increase in the absorption peak (Figure 5d), indicating the gradual transformation of DASA into its blue-colored enol form. After 6 min, the maximum absorption peak recovered to 94.5% of its initial value. These experimental results confirmed the successful synthesis of a 660 nm responsive polymer.

**Figure 6:**
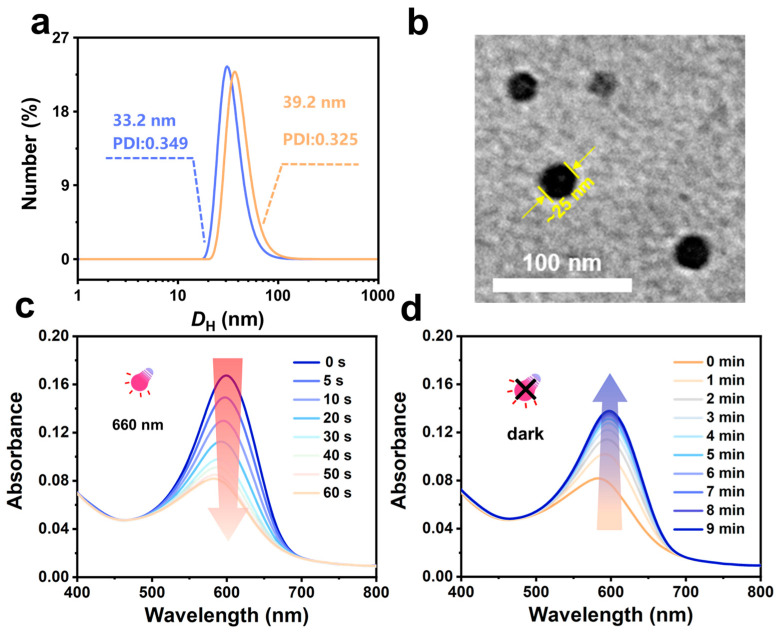
(**a**) The *D*_H_ of DASA–polymer micelles before (purple) and after (yellow) 660 nm irradiation. (**b**) The TEM image of the DASA–polymer micelle. Time-dependent absorption spectral changes of the DASA–polymer micelle in H_2_O under 660 nm irradiation (150 mW cm^−2^, (**c**)) and its subsequent thermal recovery in the dark (**d**).

Given that biological tissues have a large amount of water, we next investigated the photoresponsivity of DASA–polymer in an aqueous environment. Through self-assembly in water, the hydrodynamic diameter (*D*_H_) of the DASA–polymer is about 33 nm (Figure 6a). To verify the formation of micelles, TEM observations were conducted. As shown in Figure 6b and Figure S7, the aggregates of DASA–polymer appear spherical shape in the dry state, signifying the formation of micelles. Moreover, these micelles demonstrate an average diameter of 25 nm (Figure S8), which is smaller than the *D*_H_ observed in water. This reduction of size can be attributed to the evaporation of water within the micelles during the sample preparation process.

Figure 6c shows the absorption spectral changes of DASA–polymer micelles in H_2_O under 660 nm irradiation. As can be seen, the absorption peak in H_2_O is also broad and the maximum peak is at 600 nm. After 660 nm irradiation, similar to the changes observed in THF, the peak value of the maximum absorption peak gradually decreases with increasing irradiation time and begins to level off after 30 s. Figure 6d shows the time-dependent change in the absorption spectra of the DASA–polymer micelles after irradiation under dark conditions. It can be observed that the maximum absorption peak of micelles gradually recovers over time and begins to level off after 3 min, reaching 67.7% of the initial value after 9 min. In our previous study, the grafted DASA units in the designed green-light-responsive polymer spontaneously transformed into the cyclopentenone form in H_2_O, resulting in premature drug release [4]. In contrast, the DASA-based red-light-responsive polymer micelles designed and prepared in this study were less affected by water and were able to partially return to the hydrophobic state after 660 nm irradiation, thereby greatly avoiding premature drug release.

Certainly, it is insufficient to solely compare our previous work. The high water stability is also reflected by the ability of the DASA–polymer to rapidly revert back to its initial hydrophobic state after exposure to light. This is crucial as a slower recovery rate can lead to drug leakage and reduced controllability of drug release. Hence, we compared our results to previous reports from the perspective of thermal recovery half-life in water. The findings are summarized in Table S2. Compared to prior studies, our DASA–polymer exhibited the shortest thermal recovery time (only 1 min). These results fully showcase the remarkable water stability of the amphiphilic polymer we designed.

Last but not least, the photofatigue performance in water is also worth noting. This is because organic photoresponsive compounds are prone to degradation under light exposure, which can compromise water stability and drug release. Therefore, we further conducted photofatigue performance tests (see Figure S9). We discovered that even after three prolonged light exposures (each lasting 60 seconds at 150 mW cm^−2^), the DASA–polymer retained excellent recovery performance in the water, indicating remarkable resistance to photofatigue. The aforementioned results unequivocally demonstrate the outstanding water stability performance of the designed DASA–polymer. This represents a further step towards the practical application of DASA-based photoresponsive polymers.

What is more, we also performed DLS measurements on DASA–polymer micelles after irradiation (Figure 6a). The observed increase in size can be attributed to the conversion of DASA units from a hydrophobic to a hydrophilic state, resulting in the expansion of the micellar cores due to the influx of water molecules. It is worth noting that the micelles experience swelling rather than complete dissolution, which could be attributed to the limited incorporation of DASA units within a single polymer chain, insufficient to induce complete dissolution of the DASA–polymer micelles.

**Figure 7:**
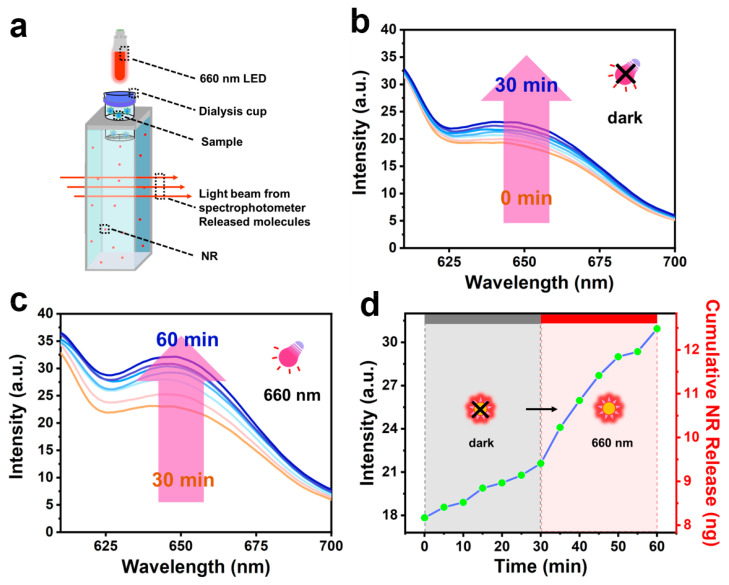
(**a**) The setup used to detect released NR molecules upon 660 nm irradiation. Emission spectral changes (*λ*_ex_ = 550 nm) over time from the bottom polymer solution of NR-loaded DASA–polymer nanovectors using the MINI dialysis device: (**b**) kept in the dark; (**c**) 660 nm irradiation. The emission spectra were recorded once every 5 min. (**d**) Plots of emission at 650 nm and cumulative NR release vs. time.

After 660 nm irradiation, the expansion of micelles should facilitate the release of loaded drugs. To verify our hypothesis, we set up a device for detecting the drug release behavior of the DASA–polymer nanovector using dialysis cups and cuvettes (Figure 7a). The mini dialysis cup (MWCO 3500) on the top of the device was used to hold the solution of NR-loaded DASA nanovector (0.5 mL), while the solution of F-127 polymer micelles (1 mg mL^−1^) was placed in the cuvette below. By tracking the fluorescence emission spectral changes in the cuvette, it can be demonstrated whether NR can be released upon 660 nm irradiation.

Firstly, the emission intensity at 650 nm (the maximum emission peak of NR) was monitored for 60 min under dark conditions. As illustrated in Figure S10a, we observed a slight increase in fluorescence intensity at 650 nm. This phenomenon can be attributed to the diffusion of NR encapsulated in the nanovectors driven by concentration gradients.

Secondly, to confirm the accelerated release using 660 nm light, the 660 nm LED light was switched on after 30 min. Figure 7 presents the corresponding results. Specifically, Figure 7b,c display the changes in emission spectra of the solution in the cuvette during the dark period (0–30 min) and the illuminated period (30–60 min), respectively. Under dark conditions, we found that the nanovector released the NR molecules very slowly, while under irradiation conditions, the release rate of NR was significantly increased. To better compare the difference in NR release rates under these two conditions, we also plotted the fluorescence emission intensity at 650 nm as a function of time, and the results are shown in Figure 7d. Notably, the cumulative amount of NR release is determined using the standard calibration curve presented in Figure S11. It can be seen that after 660 nm exposure, the release rate of NR was significantly accelerated, and the difference in fluorescence intensity or cumulative NR release between before and after light exposure continued to increase with the increase in total irradiation time.

Finally, to ensure that the 660 nm irradiation does not induce emission from other substances in the sample, a control experiment was conducted in the absence of NR. As shown in Figure S10b, for the NR-free DASA–polymer nanovector, the fluorescence intensity at 650 nm also exhibited a little increase regardless of illumination due to unknown factors. However, within the 30–60 min range, the increase in intensity was not as pronounced as depicted in Figure 7d. The above experimental results confirm that the designed DASA–polymer can be used for drug release under 660 nm irradiation.

## 4. Conclusions

In conclusion, we have demonstrated the design and synthesis of a red-light-responsive polymer by incorporating DASA units into an amphiphilic block polymer. This photoresponsive polymer can self-assemble into micelles in H_2_O (about 33 nm hydrodynamic diameter), during which the hydrophobic model drug molecules can be trapped in their hydrophobic core. Our results demonstrate that the constructed red-light-responsive nanovector was able to release trapped cargo under 660 nm. Given the strong designability, tunable response wavelength, heavy metal-free nature, and high water stability of DASA, we hope that this work will encourage more researchers to use DASA in the development of next-generation photoresponsive polymer nanomedicines, further advancing their practical applications. Further works, such as the construction of red-light-responsive DASA–polymer vesicles which have large drug-loading capacity, are ongoing in our labs.

## Data Availability

All data are contained within the article and the Supplementary Material or available upon email request from the authors.

## Supplementary Materials

The following supporting information can be downloaded at: https://www.mdpi.com/article/10.3390/polym15112489/s1, Figure S1: ^1^H (top), ^13^C (middle), and ^19^F NMR (bottom) spectra of the compound (**1**) (pentafluorophenyl methacrylate) in CDCl_3_ (25 °C, 400 MHz); Figure S2: ^1^H NMR (25 °C, 400 MHz) spectra of the compound (**2**) in DMSO (top) and compound (**3**) in CDCl_3_ (bottom), respectively; Figure S3: ^1^H NMR (top, CDCl_3_, 25 °C, 400 MHz) and mass (bottom) spectra of the compound (**4**); Figure S4: ^1^H NMR (top, CDCl_3_, 25 °C, 400 MHz) and mass (bottom) spectra of the compound (**5**); Figure S5: ^1^H NMR (25 °C, 400 MHz) spectrum of polymer (**6**) in CDCl_3_; Figure S6: ^1^H NMR (top, CDCl_3_, 25 °C, 400 MHz) and mass (bottom) spectra of the compound (**9**); Figure S7: TEM observations of the DASA–polymer aggregates at various magnifications; Figure S8: The size distribution histogram of DASA–polymer micelles unveils an average diameter of 25 nm; Figure S9: Fatigue resistance study of DASA–polymer in water (150 mW cm^-2^); Figure S10: Plots of emission at 650 nm vs time. The time periods at which the 660 nm is turned off (gray area) or turned on (pink area) are indicated. (a) NR-loaded DASA–polymer nanovectors. (b) NR-free DASA–polymer nanovectors; Figure S11: The standard linear calibration curve of NR in the solution of F-127 polymer micelles (1 mg mL^-1^ in H_2_O). (*λ*_ex_, 550 nm; *λ*_em_, 650 nm); Figure S12: The emission spectrum of the red light-LED; Table S1: GPC results; Table S2: Apparent half lifetimes to reach equilibrium in the dark at 25 °C in water.
