# Supplementary material for: A Red-Light-Responsive DASA–Polymer with High Water Stability for Controlled Release
Source: Polymers (Basel). 2023 May 28;15(11):2489. doi: 10.3390/polym15112489 (PMC10255345; doi:10.3390/polym15112489)
Supplement: Supplementary file 1 [file polymers-15-02489-s001.zip › polymers-2364225-supplementary.pdf]

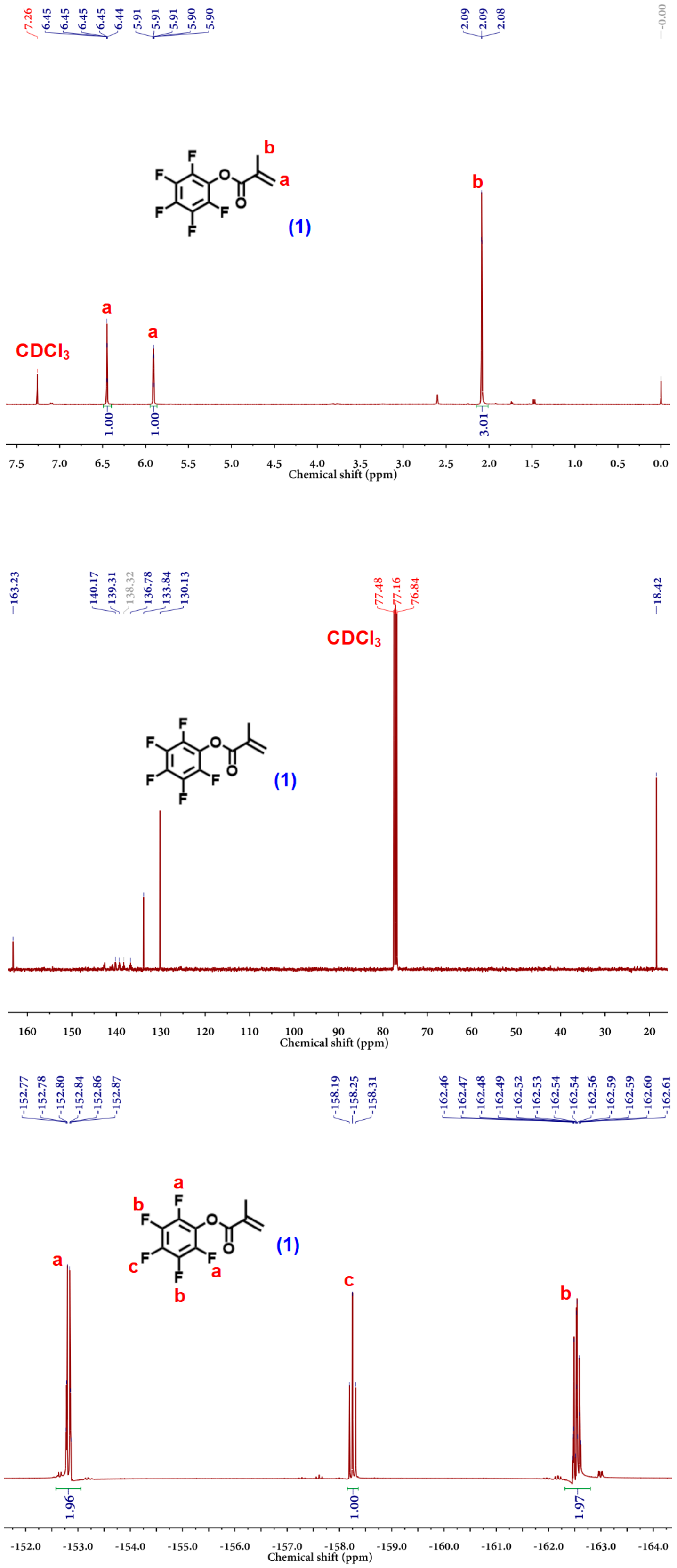

Figure S1. ^{1}H (top), ^{13}C (middle), and ^{19}F NMR (bottom) spectra of the compound (**1**) (pentafluorophenyl methacrylate) in $CDCl_3$ (25 ºC, 400 MHz).

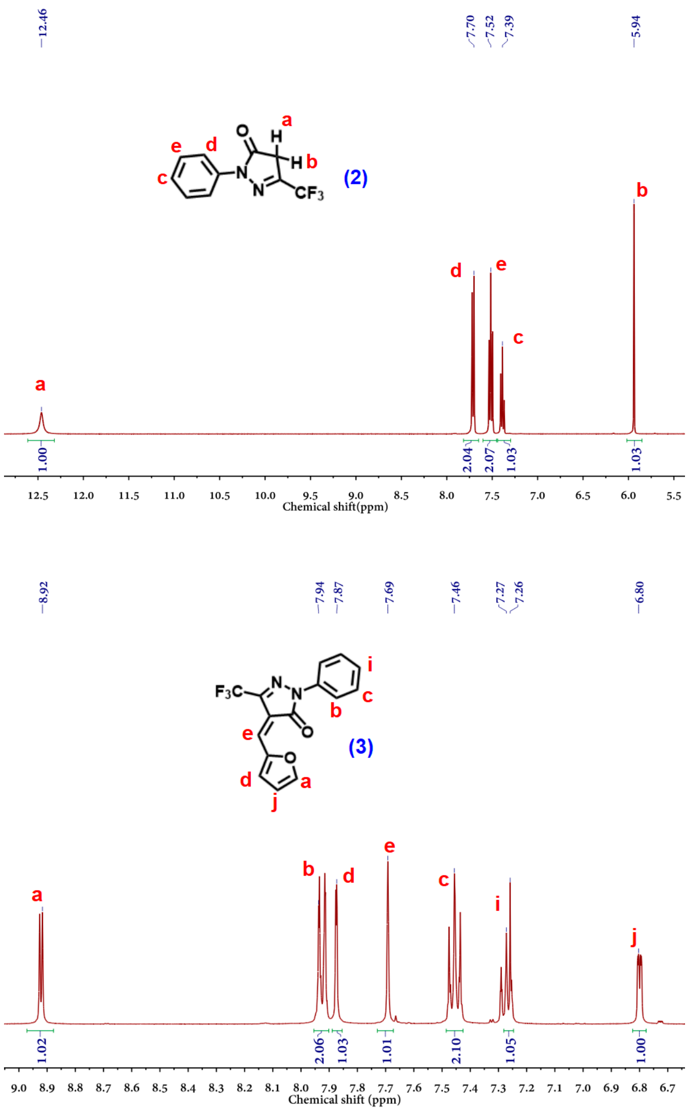

Figure S2. ^{1}H NMR (25 °C, 400 MHz) spectra of the compound (**2**) in DMSO (top) and compound (**3**) in $CDCl_3$ (bottom), respectively.

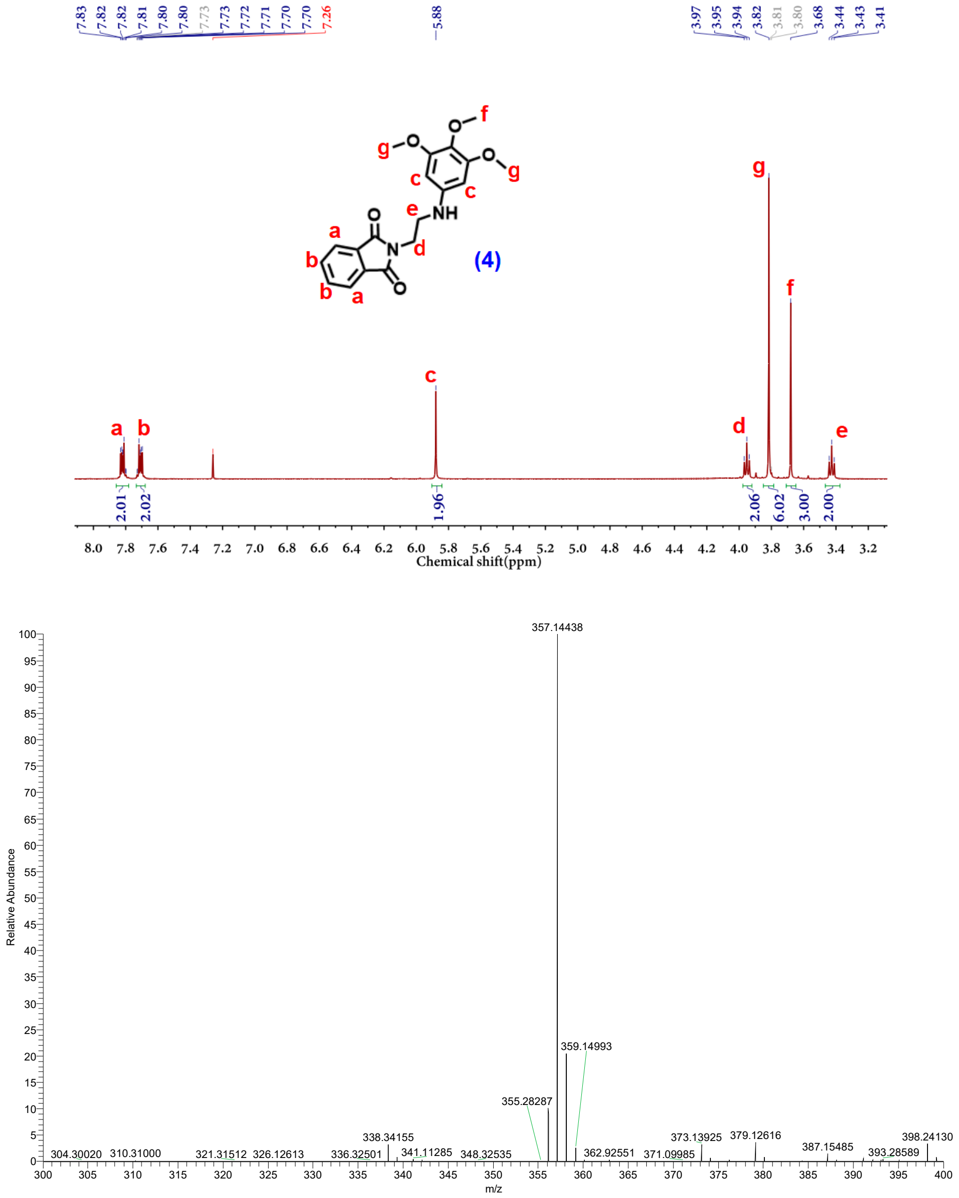

Figure S3. ^{1}H NMR (top, $CDCl_3$, 25 ºC, 400 MHz) and mass (bottom) spectra of the compound (**4**).

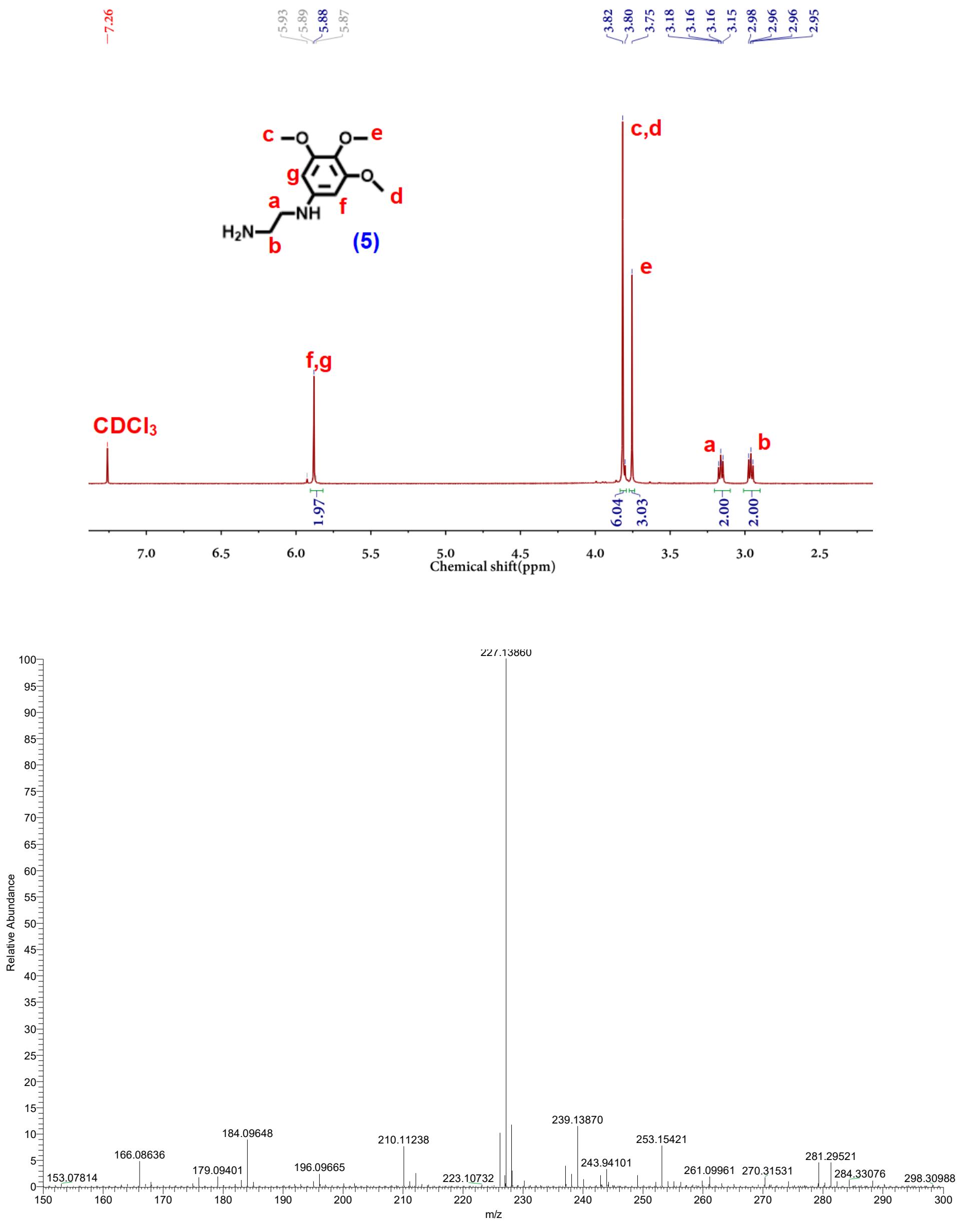

Figure S4. ^{1}H NMR (top, $CDCl_3$, 25 °C, 400 MHz) and mass (bottom) spectra of the compound (**5**).

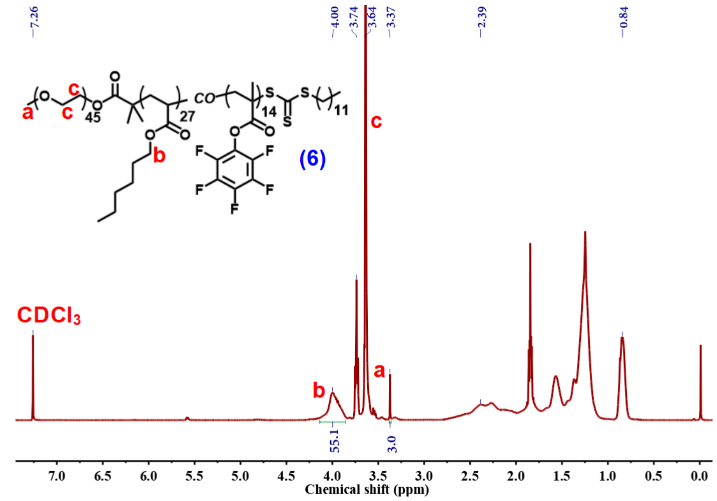

Figure S5. ^{1}H NMR (25 °C, 400 MHz) spectrum of polymer (**6**) in CDCl3.

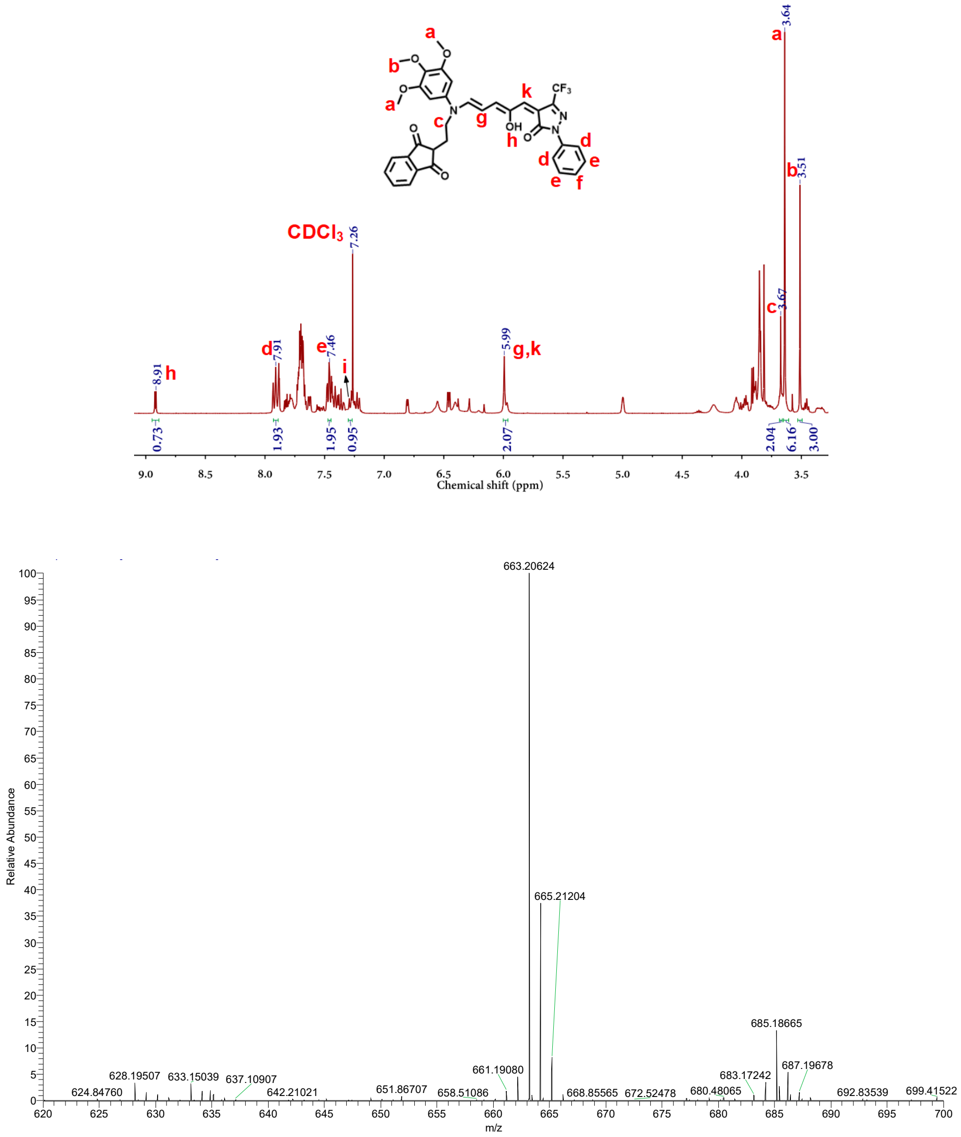

Figure S6. ^{1}H NMR (top, CDCl3, 25 °C, 400 MHz) and mass (bottom) spectra of the compound (**9**).

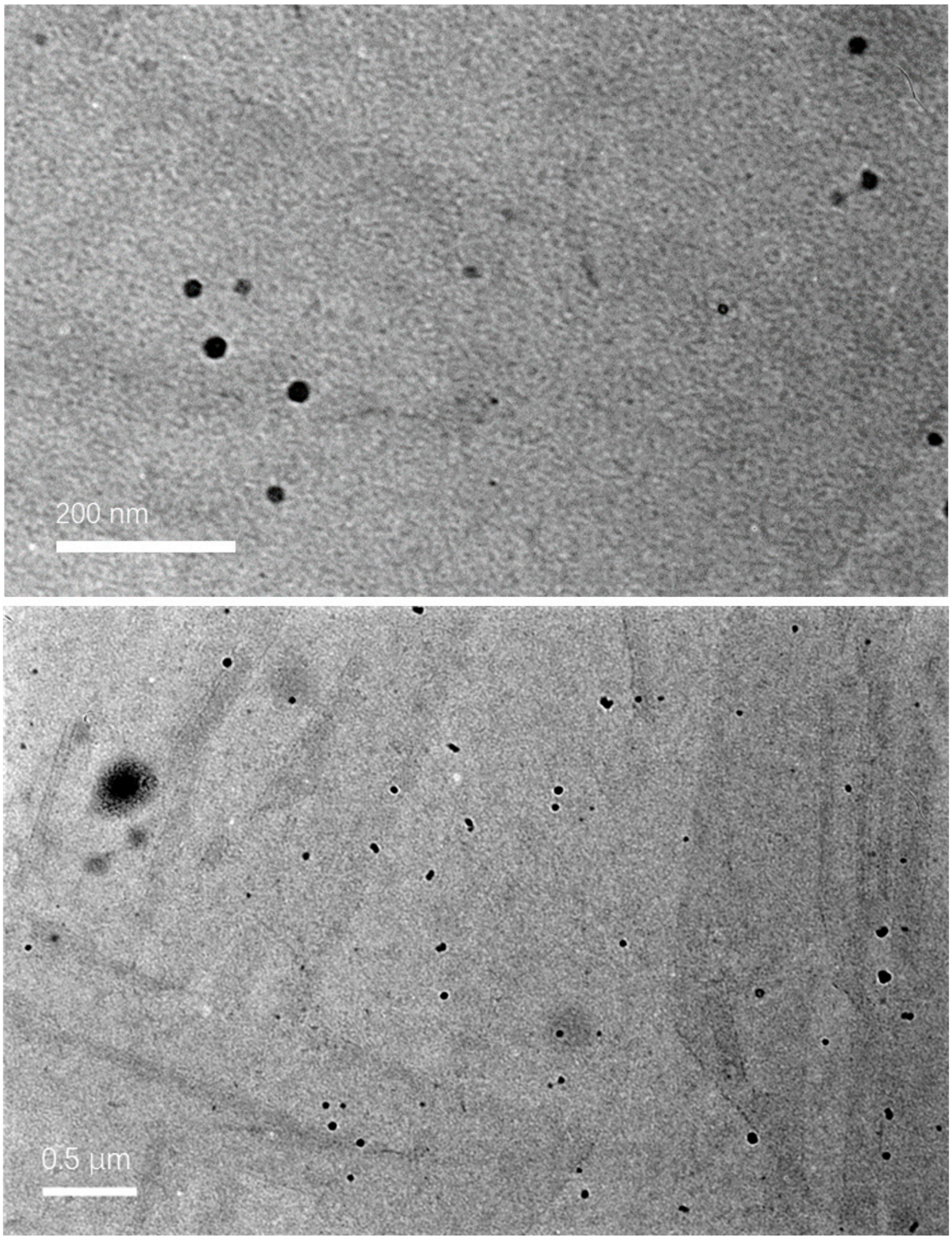

Figure S7. TEM observations of the DASA–polymer aggregates at various magnifications.

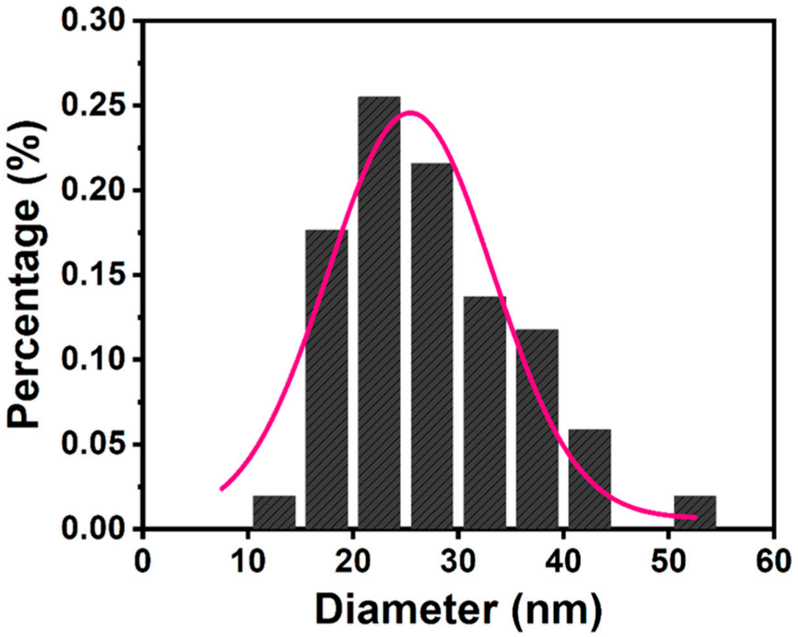

Figure S8. The size distribution histogram of DASA–polymer micelles unveils an average diameter of 25 nm.

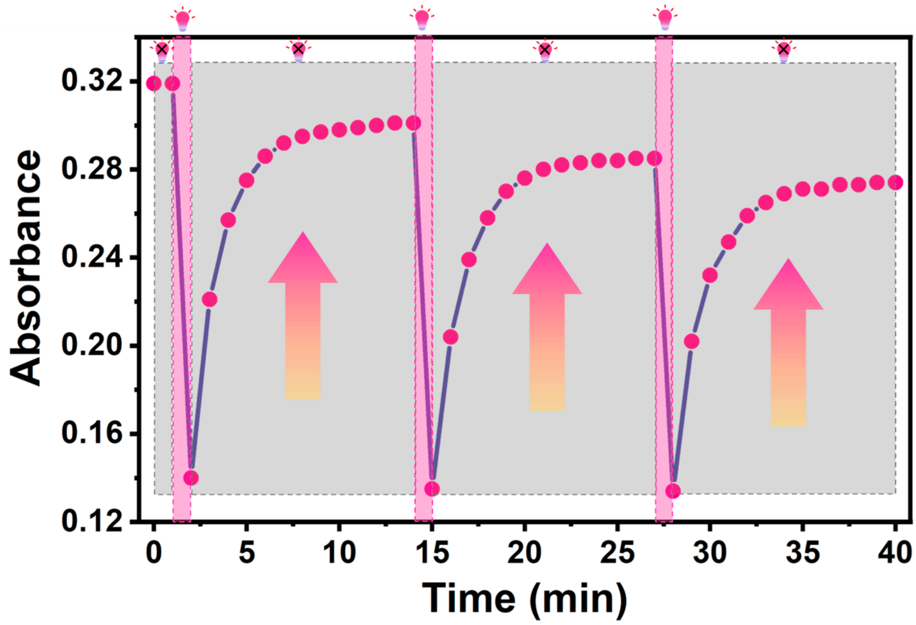

Figure S9. Fatigue resistance study of DASA–polymer in water (150 mW cm^{-2}).

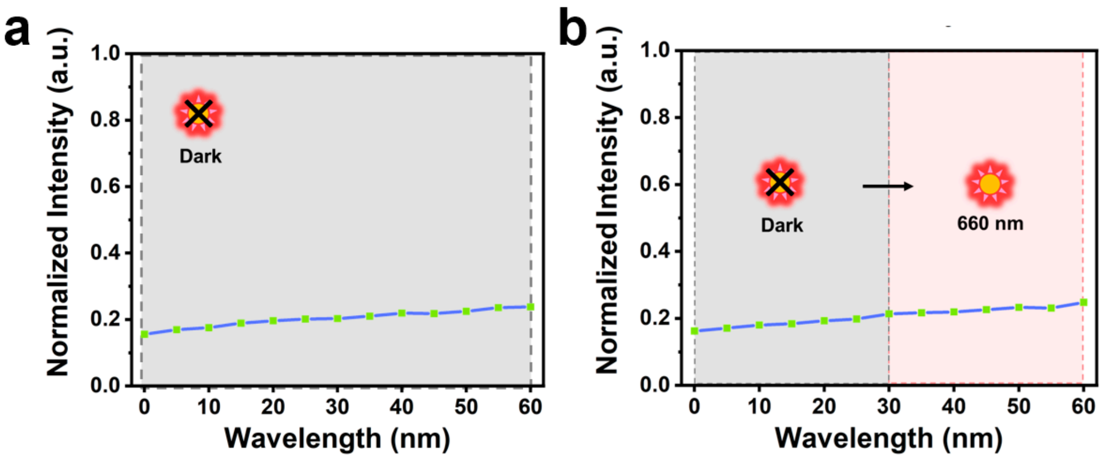

Figure S10. Plots of emission at 650 nm vs time. The time periods at which the 660 nm is turned off (gray area) or turned on (pink area) are indicated. (a) NR-loaded DASA-polymer nanovectors. (b) NR-free DASA-polymer nanovectors.

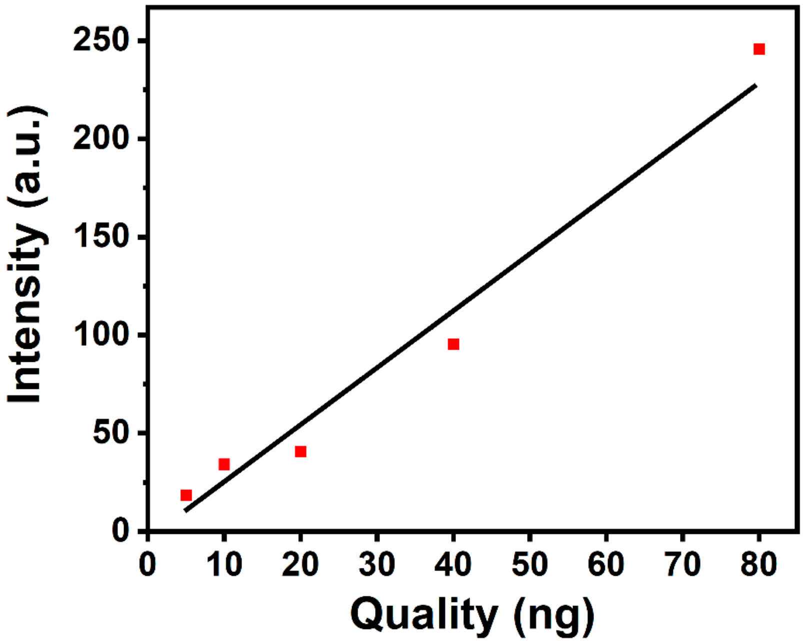

Figure S11. The standard linear calibration curve of NR in the solution of F-127 polymer micelles (1 mg mL^{-1} in H_2O). (λ_{ex}, 550 nm; λ_{em}, 650 nm).

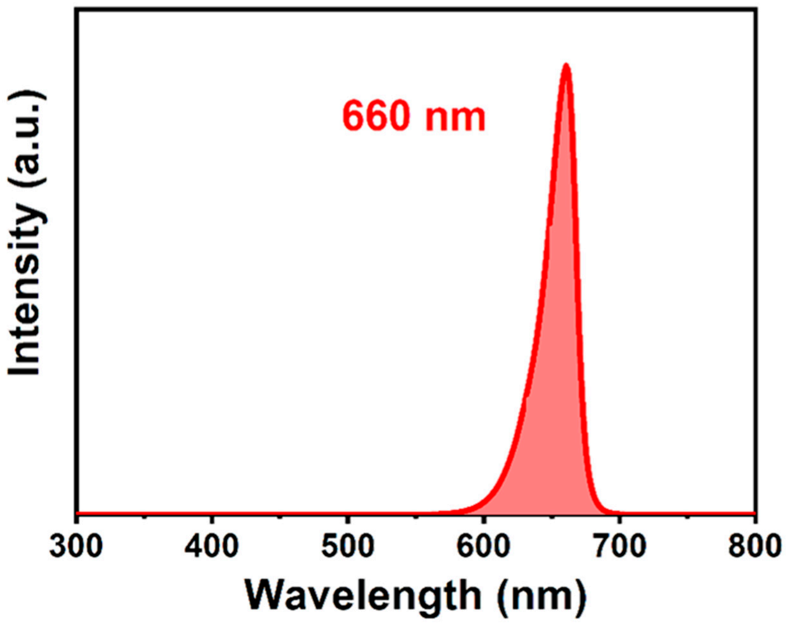

Figure S12. The emission spectrum of the red light-LED.

Table S1. GPC results.

	Mn	Mw	PDI
Polymer (**6**)	10,100	12,500	1.24
PEO_{45}-DDMAT	3,100	3,200	1.03

Table S2. Apparent half lifetimes to reach equilibrium in the dark at 25 °C in water.

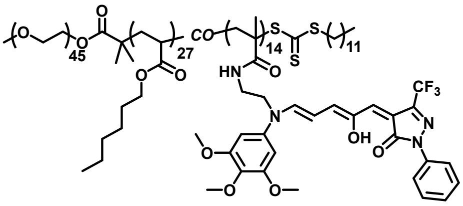

DASA-polymer

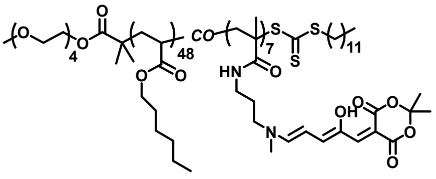

DASA ADC (PEO_{45}-*b*-P($DASA_7$-*co*-HA_{48}))

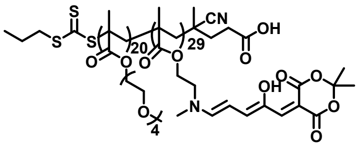

$pPEGMEMA_{20}$-*b*-$pMAEMA_{29}$ DASA

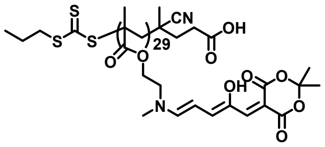

$PMAEMA_{29}$ DASA

Sample	$t_{1/2}$ (min)	Reference
**DASA-polymer**	**1**	this work
DASA ADC (PEO_{45}-*b*-P($DASA_7$-*co*-HA_{48}))	∞	Macromol. Rapid Commun. **2021**, 42, 2100318
$PMAEMA_{29}$ DASA	37	Polym. Chem. **2019**, 10, 6515
$pPEGMEMA_{20}$-*b*-$pMAEMA_{29}$ DASA	67	Macromol. Rapid Commun. **2020**, 41, 2000236
